# *Staphylococcus* species and *Mammaliicoccus* species isolated from milk and teat apex of Lacaune sheep: Occurrence of antimicrobial resistance

**DOI:** 10.3168/jdsc.2025-0931

**Published:** 2026-02-28

**Authors:** Mariluce C. Oliveira, Jennifer E. Amorim, Larissa S. Polydoro, José A. Ferronatto, Raysa B.M. Maia, Camila F. Batista, Marla Schneider, Maiara G. Blagitz, Gisele O. Souza, Marcos B. Heinemann, Alice M.M.P. Della Libera

**Affiliations:** 1 Department of Internal Medicine, Faculty of Veterinary Medicine and Animal Science, University of São Paulo, Cidade Universitária, 05508-270, São Paulo, Brazil; 2 Federal University of the Southern Border, Cidade Universitária, Realeza, Paraná, Brazil 85770-000; 3 Department of Preventive Veterinary Medicine and Animal Health, Faculty of Veterinary Medicine and Animal Science, University of São Paulo, Cidade Universitária, 05508-270, São Paulo, Brazil

## Abstract

Antimicrobial resistance is a growing concern in dairy production, with direct implications for animal health, food safety, and public health. This study aimed to characterize the phenotypic and genotypic resistance profiles of *Staphylococcus* spp. and *Mammaliicoccus* spp. isolated from milk and teat apex samples of 50 Lacaune sheep in early lactation. A total of 100 isolates (50 from milk and 50 from the teat apex) were identified using MALDI-TOF and subjected to disk diffusion tests with oxacillin (1 µg) and cefoxitin (30 µg). Molecular screening for *mecA* and *mecC* genes was performed by PCR on all isolates, including those that were phenotypically susceptible. Overall, 19% of isolates were resistant to oxacillin and 10% to cefoxitin. The *mecA* gene was detected in 2 oxacillin-resistant strains, whereas *mecC* was identified in 7 isolates, including *Staphylococcus xylosus*, *Staphylococcus saprophyticus*, and *Mammaliicoccus sciuri*. Notably, several *mecC*-positive isolates were phenotypically susceptible, highlighting the limitations of conventional methods and the risk of underdiagnosis. These findings underscore the value of molecular screening as a complementary tool for the accurate detection of methicillin resistance, particularly in NAS from small ruminants. The detection of resistance genes in isolates from both milk and the teat apex suggests that the teat apex may act as a reservoir and source of antimicrobial resistance dissemination. This study provides important insights into resistance dynamics in dairy sheep. It highlights the role of this anatomical interface in the transmission chain, emphasizing its importance in integrated surveillance efforts within the One Health framework.

## Highlights

• One Health view of methicillin resistance in Lacaune dairy ewes.
• Of the isolates tested, 19% were oxacillin resistant and 10% were cefoxitin resistant.
• *mecA* and *mecC* were found in non-*aureus* staphylococci and *M. sciuri*.
• Some *mecC*-positive isolates appeared phenotypically susceptible.
• Milk and teat apex act as reservoirs of methicillin resistance.

## Graphical Abstract

**Figure:**
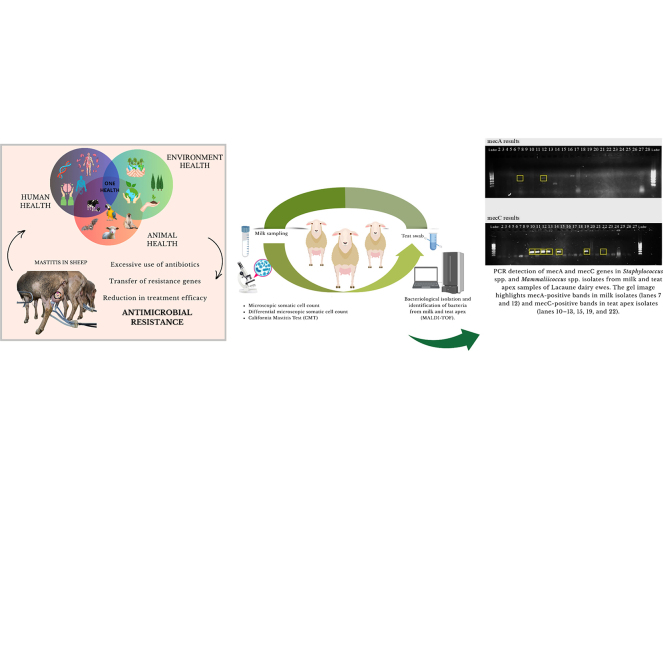
**Summary:** This graphical abstract places methicillin resistance in Lacaune dairy ewes within a One Health framework, linking mastitis, excessive antibiotic use, and the movement of resistance genes across animals, the environment, and humans. Milk samples were screened by somatic cell count and California Mastitis Test, and teat apex swabs were collected for bacteriological culture and identification of *Staphylococcus* spp. and *Mammaliicoccus* spp. using matrix-assisted laser desorption/ionization time-of-flight (MALDI-TOF). Polymerase chain reaction (PCR) detection of *mecA* and *mecC* in isolates from both milk and teat apex revealed the teat apex as an important reservoir of methicillin resistance genes on dairy sheep farms, with direct implications for mastitis control and antimicrobial stewardship. Left image: Illustration created by the authors using Canva. Middle image: Created with BioRender.com. Right image: Representative image obtained in the present study.

Antimicrobial resistance (**AMR**) is widely recognized as a critical global public health concern that has profound impacts on both human and veterinary medicine. Its rise is mainly driven by the inappropriate use of antimicrobials in clinical and agricultural settings, poor infection control, and the environmental spread of resistance genes (Pieri et al., 2020; Kasimanickam et al., 2021; Msemakweli et al.*,* 2024). In response, the World Health Organization has called for coordinated efforts under a One Health approach, integrating human, animal, and environmental health (WHO, 2022).

In this context, the dairy sheep sector plays a dual role, being a source of high nutritional value products, but also a potential reservoir of resistant bacteria. Subclinical mastitis, in particular, compromises milk quality and often requires antimicrobial therapy, increasing the risk of drug residues and the dissemination of resistance (Balthazar et al., 2017; Cannas et al., 2019; Alba et al.*,* 2019).

*Staphylococcus* spp. are the primary pathogens associated with mastitis in sheep, with NAS such as *Staphylococcus chromogenes*, *Staphylococcus epidermidis*, *Staphylococcus simulans*, and *Staphylococcus xylosus* being frequently isolated (Vanderhaeghen et al.*,* 2014; Gelasakis et al., 2015; Regecová et al.*,* 2021). Although these bacteria are part of the natural mammary gland microbiota, they may act as opportunistic pathogens under conditions that facilitate their entry into the teat canal (De Buck et al.*,* 2021). Recently, the genus *Mammaliicoccus* spp. was proposed, comprising species previously classified as NAS. Among them, *Mammaliicoccus sciuri* has been frequently recovered from subclinical mastitis cases and is known to carry resistance genes such as *mecA* and *mecC* (Madhaiyan et al.*,* 2020; Moura et al.*,* 2023).

Although methicillin is not commonly used in veterinary mastitis treatment (Harrison et al.*,* 2014), the presence of methicillin-resistant staphylococci in raw milk and dairy products raises substantial public health concerns (Cicconi-Hogan et al.*,* 2014; Giacinti et al., 2017; Khemiri et al.*,* 2018; Tohoyessou et al.*,* 2020; Freitas Ribeiro et al., 2020; Shrestha et al.*,* 2021). Nelli et al. (2022) reported the presence of *mecA* in 89% of isolates suspected of methicillin resistance from goat milk samples, whereas Moura et al. (2023) highlighted the frequent isolation of *M. sciuri* from ewes with subclinical mastitis, reinforcing its role as an emerging resistant pathogen.

Additionally, the teat apex represents a critical niche for the colonization and persistence of resistant bacteria. Constantly exposed to environmental contaminants and milking equipment, this region may act as a key site for the exchange and maintenance of resistance genes, reinforcing its relevance in surveillance strategies within the One Health framework (Zadoks et al.*,* 2002; Braem et al.*,* 2013).

This study aimed to characterize the antimicrobial resistance profile of *Staphylococcus* spp. and *Mammaliicoccus* spp. isolated from both milk and teat apex samples of lactating ewes with subclinical mastitis, focusing on phenotypic resistance to oxacillin and cefoxitin, as well as the genotypic detection of the *mecA* and *mecC* resistance genes.

In this study, we evaluated 100 isolates of *Staphylococcus* spp. and *Mammaliicoccus* spp. (50 from milk and 50 from teat apex swabs) selected from a previous CEUA (Ethics Committee on Animal Use)-approved investigation conducted as part of a graduate thesis at the Faculty of Veterinary Medicine and Animal Science, University of São Paulo (FMVZ/USP). Samples were collected from 50 lactating Lacaune ewes housed on a semi-confined dairy farm in Chapecó, Santa Catarina, Brazil, during the first month of lactation, under mechanical milking. Sampling procedures were standardized and performed at the animal level. Milk was aseptically collected from each mammary half after discarding the first streams and disinfecting the teats with an antiseptic solution (2 milk samples per ewe per sampling occasion). Teat apex samples were obtained from each teat using sterile swabs moistened with sterile saline, as described by De Vliegher et al. (2003; 2 teat apex swabs per ewe per sampling occasion). Overall, 597 samples (milk and teat apex) were obtained, yielding 206 isolates from milk and 669 isolates from teat apex swabs. From this isolate collection, the subset of 100 isolates included in the present study was selected based on relative frequency, epidemiological importance, and relevance for antimicrobial resistance surveillance, aiming to represent the most frequent and epidemiologically relevant taxa while balancing the sampling site (milk and teat apex) and prioritizing *Staphylococcus* spp. and *Mammaliicoccus* spp. All isolates were identified by MALDI-TOF and stored in cryovials at −80°C until analysis.

The selection of isolates was based on (1) high relative prevalence at each site, (2) epidemiological relevance to ovine mastitis, and (3) recurrence of the same species across different animals. Before analysis, isolates were thawed, vortexed, and 100 μL were transferred into sterile tubes with 3 mL of brain heart infusion (**BHI**), incubated at 37°C for 24 h, then streaked on blood agar and incubated again at 37°C for 24 h.

Antimicrobial susceptibility was assessed via disk diffusion. Isolates were standardized to a 0.5 McFarland turbidity in 0.85% saline, plated on Mueller–Hinton agar, and tested with oxacillin (1 μg) and cefoxitin (30 μg) disks. After 24 h at 37°C, inhibition zones were measured and interpreted as susceptible, intermediate, or resistant, following CLSI (2021) guidelines.

Genomic DNA was extracted using a boiling method adapted from Fan et al. (1995). Colonies were suspended in TE buffer (10 m*M* Tris-HCl, 5 m*M* EDTA, pH 8.0), boiled at 90°C for 15 min, then frozen at −20°C for 20 min. After thawing, the samples were vortexed and centrifuged at 1,400 × *g* for 5 min at 4°C. The supernatant was transferred to clean microtubes and stored at −20°C.

Detection of the *mecA* and *mecC* genes was performed using PCR protocols adapted from Mehrotra et al. (2000). Each 15-μL reaction contained GoTaq Green Master Mix (Promega), ultrapure water, primers (0.3 μL each), and 2 μL of DNA. Primer sequences were as follows: *mecA*, forward 5′-ACTGCTATCCACCCTCAAAC-3′, reverse 5′-CTGGTGAAGTTGTGTGTGTAATCTGG-3′; *mecC*, forward 5′-CATTAAAATCAGAGCGAGGC-3′, reverse 5′-TGGCTGAACCCATTTTTGAT-3′. Thermal cycling conditions for *mecA* were 94°C for 5 min; 35 cycles of 94°C for 2 min, 57°C for 2 min, and 72°C for 1 min; and final extension at 72°C for 7 min. For *mecC*, conditions were 94°C for 5 min; 35 cycles of 94°C for 30 s, 52°C for 30 s, and 72°C for 1 min; and final extension at 72°C for 10 min. All isolates were screened regardless of phenotypic resistance to account for heteroresistance or cryptic resistance.

The PCR products were resolved by electrophoresis on 1.5% agarose gels prepared in 0.5× Tris–borate–EDTA buffer, stained with gel dye (1 μL/10 mL), and visualized under UV light. Negative controls (TE buffer) and positive controls (*Staphylococcus aureus* ATCC 25923) were included. Gels were run at 90 V for 30 min and cooled before visualization.

Statistical analyses included descriptive statistics for resistance profiles and gene detection, and Fisher's exact test for categorical associations. To assess the effects of predictor variables on ordinal resistance outcomes (susceptible < intermediate < resistant), an ordinal logistic regression (proportional odds model) was applied using the polr function (MASS package, R software version 4.5.0, R Foundation for Statistical Computing). Significance was set at *P* < 0.05. Resistance patterns were visualized in a heatmap using the packages readxl, dplyr, ggplot2, forcats, MASS, and reshape2 in R.

A total of 875 bacterial isolates were obtained from 597 samples (milk and teat apex) collected from lactating Lacaune ewes. Among these, 638 isolates (157 from milk and 481 from teat apex) were identified as *Staphylococcus* spp. using MALDI-TOF MS, representing 76.21% of all isolates. *Staphylococcus xylosus* was the most prevalent species in both milk (23.5%) and teat apex (58.4%) samples. Other frequently identified species included *S. simulans*, *S. chromogenes*, *Staphylococcus saprophyticus*, and *S. equorum*, indicating a diverse staphylococcal microbiota associated with both anatomical sites (Zafalon et al.*,* 2017; Eriksson et al.*,* 2013; Gelasakis et al.*,* 2015; Table 1).

**Table 1:** Distribution of *Staphylococcus* spp. and *Mammaliicoccus* spp. bacterial strains isolated from milk and the teat apex samples of Lacaune dairy ewes; the table presents the frequency and diversity of the identified isolates, providing a detailed characterization of the species present in the different samples^1^

Species^2^	Milk		Teat apex		Total
	AB	RN (%)	NA	N (%)	
*M. sciuri*	9	5.73	27	5.61	36
*S. chromogenes*	23	14.65	5	1.03	28
*S. equorum*	20	12.74	32	6.65	52
*S. xylosus*	37	23.57	281	58.42	318
NAS	20	12.74	—	—	20
*S. simulans*	30	19.11	5	1.03	35
*S. auricularis*	1	0.64	—	—	1
*M. lentus*	1	0.64	3	0.62	4
*S. microti*	2	1.27	—	—	2
*S. epidermidis*	4	2.55	2	0.41	6
*S. saprophyticus*	4	2.55	112	23.28	116
*S. aureus*	6	3.82	4	0.83	10
*S. nepalensis*	—	—	4	0.83	4
*S. warneri*	—	—	6	1.25	6
Total	157	100	481	100	638

1 AB = absolute numbers; represents the total count of isolates for each species identified in each niche evaluated (milk and teat apex). RN = relative numbers; corresponds to the percentage of microorganisms of the same species in relation to the total bacterial population identified within each niche (milk and teat apex). NA = total number of teat apex samples analyzed.

2 Species refers to the microorganism isolated and identified from the samples analyzed.

Based on the subset of 100 isolates (50 from milk and 50 from teat apex) selected for antimicrobial resistance, the oxacillin was observed in 16 milk isolates (32%) and in 3 teat apex isolates (6%). Resistance to cefoxitin was detected in 3 milk isolates (6%) and in 7 teat apex isolates (14%). The frequency of isolates exhibiting intermediate susceptibility was low for both antimicrobials. These results reveal a higher prevalence of oxacillin resistance in milk isolates and greater cefoxitin resistance in teat apex isolates, underscoring the heterogeneous distribution of resistance across anatomical niches (CLSI, 2021; Table 2).

**Table 2:** Number of species used in this study and the antibiogram and PCR of *Staphylococcus* spp. and *Mammaliicoccus* spp. obtained from milk and teat apex samples of Lacaune dairy sheep; the table represents the number of isolates classified as resistant or intermediate to oxacillin and cefoxitin in the disk diffusion test and PCR detection of *mecA* and *mecC* resistance genes^1^

Species^2^	Milk				Teat apex			
	NIT	Oxacillin	Cefoxitin	PCR	NIT	Oxacillin	Cefoxitin	PCR
*S. xylosus*	11	5 R	1 R	—	19	2 I	3 R	4 *mecC*
		2 I						
*S. aureus*	6	4 R	1 I	1 *mecA*	4	—	—	—
*S. simulans*	8	—	1 R	—	2	—	—	—
*S. chromogenes*	7	—	1 R	—	3	—	1 R	1 *mecC*
NAS	8	3 R	—	—	—	—	—	—
		1 I						
*S. equorum*	4	1 R	—	—	7	—	1 R	—
*M. sciuri*	4	2 R	—	1 *mecA*	5	—	1 R	1 *mecC*
*S. epidermidis*	1	1 R	—	—	1	—		
*S. saprophyticus*	1	—	—	—	9	3 R	1 R	1 *mecC*
Total	50 isolates	16 R	3 R	2 *mecA*	50 isolates	3 R	7 R	7 *mecC*
		3 I	1 I			2 I		

1 NIT = number of isolates tested. R refers to the antibiotic-resistant microorganism tested, and I refers to the microorganism intermediately resistant to the antibiotic tested.

2 Species refers to the microorganism isolated and identified from the samples analyzed.

The PCR analysis revealed the presence of the *mecA* gene in 2 milk isolates resistant to oxacillin (*S. aureus* and *M. sciuri*). The *mecC* gene was detected in 7 isolates from the teat apex, 5 of which exhibited phenotypic resistance to cefoxitin, whereas 2 were classified as phenotypically susceptible. The *mecC*-positive isolates included 4 *S. xylosus*, 1 *S. chromogenes*, 1 *S. saprophyticus*, and 1 *M. sciuri*, highlighting the widespread presence of methicillin resistance determinants among NAS and *Mammaliicoccus* spp. in dairy sheep environments (Paterson, 2020; Moura et al.*,* 2023).

A significant association was identified between sample origin and oxacillin resistance (*P* = 0.0012), with higher resistance observed in milk isolates. No association was detected for cefoxitin resistance (*P* = 1.0). The presence of *mecA* was significantly associated with oxacillin resistance (*P* = 0.017), whereas *mecC* was significantly associated with resistance (*P* = 0.044), and regression analyses further supported its strong association with cefoxitin resistance.

Ordinal logistic regression further supported these findings. Isolates from the teat apex had significantly lower odds of resistance to oxacillin (odds ratio [**OR**] = 0.0938; *P* = 0.0026), whereas the presence of *mecC* increased the odds of resistance to both antimicrobials (Figure 1). For cefoxitin, *mecC* was strongly associated with resistance (OR = 55.3; *P* < 0.001). Although *mecA* showed a substantial effect (β = 17.227), its low frequency limited model accuracy.

**Figure 1:**
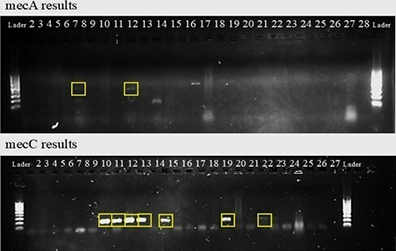
Polymerase chain reaction detection of *mecA* and *mecC* genes in *Staphylococcus* spp. and *Mammaliicoccus* spp. isolates from milk and teat apex samples of Lacaune dairy ewes. The gel image highlights *mecA*-positive bands in milk isolates (lanes 7 and 12) and *mecC*-positive bands in teat apex isolates (lanes 10–13, 15, 19, and 22). Polymerase chain reaction results for the *mecA* gene in milk samples: detected in lanes 7 (*S. aureus*) and 12 (*M. sciuri*). Polymerase chain reaction results for the *mecC* gene in teat apex samples: detected in lanes 10 (*S. xylosus*), 11 (*M. sciuri*), 12 (*S. xylosus*), 13 (*S. xylosus*), 15 (*S. chromogenes*), 19 (*S. xylosus*), and 22 (*S. saprophyticus*).

Phenotypic and genotypic correlations revealed inconsistencies; 69% of the tested isolates were susceptible to both antimicrobials, whereas 5% exhibited cross-resistance. Among *mecC*-positive isolates (n = 7), 5/7 (71.4%) were phenotypically resistant to cefoxitin by disk diffusion, whereas 2/7 (28.6%) were classified as cefoxitin-susceptible, indicating incomplete phenotypic expression of *mecC* in this dataset. *mecC* was detected only in teat apex isolates, and species-level interpretation is limited by small numbers; however, *mecC*-positive *S. xylosus* showed cefoxitin resistance in 3/4 isolates, whereas the *mecC*-positive *M. sciuri* isolate was phenotypically susceptible (Table 2). This discordance may reflect heteroresistance or subthreshold gene expression. All isolates were screened by PCR regardless of phenotypic susceptibility to cefoxitin to account for heteroresistance or cryptic resistance (Argudín et al.*,* 2011; Magiorakos et al.*,* 2012; Mendonça et al.*,* 2012; Ruegg et al.*,* 2015). These findings reinforce the importance of combining molecular and phenotypic methods for accurate resistance surveillance in ovine milk production systems.

The detection of *mecA* and *mecC* in *M. sciuri*, including one isolate from milk and another from the teat apex, respectively, is particularly noteworthy. These isolates showed corresponding phenotypic resistance, suggesting a potential role of *M. sciuri* as a reservoir for methicillin resistance genes in ovine herds. Although *M. sciuri* has been reported in cattle and buffalo (Harrison et al.*,* 2014; Khazandi et al.*,* 2018; El-Ashker et al.*,* 2020), reports in sheep remain scarce.

Although *S. aureus* was less prevalent (10 isolates; 6 in milk, 4 in teat apex), 4 of the milk isolates exhibited oxacillin resistance, and one carried *mecA*, raising concerns regarding zoonotic transmission and highlighting the need for continuous monitoring, especially in raw milk and artisanal dairy contexts (Giacinti et al.*,* 2017). Despite pasteurization inactivating viable pathogens, resistance genes may remain detectable after heat treatment and could persist in the food chain, particularly in regions where consumption of unpasteurized dairy products is common [Devirgiliis et al., 2011; EFSA Panel on Biological Hazards (BIOHAZ), 2021; James et al., 2021; Sievers et al., 2025].

A limitation of this study is that antimicrobial susceptibility testing was performed in a purposively selected subset of isolates rather than in the full collection recovered from the flock. Therefore, although the selection strategy aimed to represent the most frequent and epidemiologically relevant taxa across both anatomical sites, the results should be interpreted as a targeted screening and may not fully capture the heterogeneity of species distribution and resistance profiles present in the flock.

Additionally, udder health and follow-up data (e.g., somatic cell count and clinical outcomes) were not recorded for the tested isolates, precluding correlation analyses between resistance profiles and clinical parameters.

The higher risk of resistance in milk isolates could be influenced by selective pressure associated with mastitis treatment practices (e.g., intramammary therapy or systemic antimicrobial treatments). However, farm-level antimicrobial use data were not available for this flock, and therefore the observed differences in resistance between milk and teat apex isolates cannot be conclusively attributed to treatment-related selective pressures.

In summary, the results demonstrate that both milk and teat apex represent relevant ecological niches for resistant staphylococci in dairy sheep. The higher risk of resistance in milk isolates highlights that resistance patterns may differ between anatomical niches, whereas the teat apex may serve as a colonization site for future intramammary infections. These findings emphasize the complexity of AMR dynamics in dairy production and reinforce the necessity for integrated surveillance strategies that consider anatomical origin, species diversity, and genotypic profiles.

The presence of *Staphylococcus* spp. and *Mammaliicoccus* spp. in milk and teat apex samples highlights the complex microbial ecosystem of the ovine mammary gland and its potential role as a reservoir for antimicrobial resistance genes. The detection of *mecA* and *mecC* genes, including in phenotypically susceptible isolates, underscores the importance of integrated surveillance approaches that combine phenotypic and molecular methods. Given the relevance of dairy sheep production in agribusiness and the risk of gene dissemination, particularly through raw milk and its derivatives, continued monitoring is crucial. Future studies focusing on β-lactam resistance mechanisms in NAS may support the development of more targeted treatment strategies for mastitis and reduce the indiscriminate use of antimicrobials. Altogether, the findings support the adoption of a One Health approach and emphasize the need for robust antimicrobial resistance monitoring across the dairy production chain.
